# Vulnerable Populations Exposed to Lead-Contaminated Drinking Water within Houston Ship Channel Communities

**DOI:** 10.3390/ijerph16152745

**Published:** 2019-08-01

**Authors:** Garett Sansom, Leslie Cizmas, Kathleen Aarvig, Benika Dixon, Katie R. Kirsch, Anjali Katare, Lindsay Sansom

**Affiliations:** 1 Department of Environmental and Occupational Health, Texas A&M School of Public Health, 1266 TAMU, College Station, TX 77843, USA; 2 Department of Epidemiology and Biostatistics, Texas A&M School of Public Health, 1266 TAMU, College Station, TX 77843, USA; 3 Department of Landscape Architecture and Urban Planning, Texas A&M University, 400 Bizzell St., College Station, TX 77840, USA; 4 Department of Geography, Texas A&M University, 400 Bizzell St., College Station, TX 77840, USA

**Keywords:** lead contamination, drinking water, vulnerable communities

## Abstract

Recent events have drawn increased attention to potential lead exposures from contaminated drinking water. Further, homes with older infrastructure are at greatest risk due to the presence of the disinfectant chemical chloramine, which can leach lead from older pipes. There is a growing need to determine the extent of lead leaching especially within vulnerable communities and homes with children. This pilot study collected survey data and performed lead analysis on drinking water in the small community of Manchester in Houston, TX. Manchester is characterized by industrial sites, flooding, and a low socioeconomic population. Surveys and water analyses were completed on randomly selected homes (N = 13) and documented perceptions of participants on their drinking water regarding presence and concentration of lead. Lead was discovered in 30.8% of homes ranging from 0.6 to 2.4 (µg/L), all below the US Environmental Protection Agency action level of 15 ppb, but above the water standard goals. These findings further suggest that contaminated water is a broad issue requiring concerted efforts to ensure the health of US residents.

## 1. Introduction

Lead exposure has been associated with a wide range of adverse health effects in humans, including gastrointestinal disturbances, decreased neurological functioning, heart disease, and kidney disease [1,2]. Though naturally present in the environment, the vast majority of lead in soil and dust is attributed to anthropogenic activities such as the use of leaded paint and gasoline [3]. Lead may be introduced into the human body through inhalation, ingestion of contaminated food or water, and dermal absorption. The rate of lead absorption in humans differs by route of exposure, with the greatest uptake occurring in the gastrointestinal tract after ingestion, followed by inhalation, and lastly dermal contact [1,3].

Risk and severity of associated health outcomes vary widely by levels of lead exposure, age, and life stage. Children are most vulnerable due to greater fractional absorption of ingested lead and greater intake on a body–weight basis resulting in cognitive and behavioral abnormalities [4]. As their organ systems are not yet fully developed, the rate of lead absorption in the gastrointestinal tract of children is approximately 50% greater than adults [5]. Susceptibility to the potential harms of ingested lead is also greater among developing young children. Lead exposures sufficient to raise blood lead levels to 5 µg/dl among children can adversely impact cognitive ability [3,6,7].

Populations at risk of increased lead exposure include low-income, minority status, residence in an urban area, living in close proximity to a high-volume roadway, and/or residing in a building constructed prior to 1978 [1,3]. Majority–minority neighborhoods that shoulder an undue burden of industrial pollution, namely environmental justice communities, are similarly at greatest risk of lead poisoning [8,9,10]. Residents of older housing may experience greater exposure due to the presence of deteriorating lead-based paint and antiquated lead plumbing infrastructure [4,10,11,12]. Further, individuals of low socioeconomic status are at a greater risk of developing nutritional deficiencies, which has been associated with a five-fold increase in risk of lead poisoning [13].

While exposure may occur through a variety of sources, drinking water can make up 20% or more of a person’s total exposure [14,15]. The most common cause for the presence of lead in drinking water is the use of a combination of chloramines and chlorine during the water purification process and lead piping [16,17]. Large-scale installation of lead pipes in the United States (US) began in late 1800s, and by 1900, more than 70% of cities with populations greater than 30,000 people had installed lead water lines. In 1930, local and state governments began prohibiting or limiting the use of lead in water systems [18]. Residential neighborhoods, especially if developed before the authorization of Safe Drinking Water Act (SDWA) in 1986, are expected to have lead pipe water distribution systems [19]. In the early 20th century, the US began treating the drinking water via chlorination to combat water-borne diseases [20]. By 1998, recognition of the potential carcinogenic, neurotoxic, and teratogenic effects of some chlorine-based disinfectant byproducts prompted the EPA to support the use of alternative chemical water treatments [21]. As a result, many municipal water treatment facilities switched from using chlorine to chloramine, a group of compounds containing chlorine and ammonia [22]. Although chloramines produce fewer by-products, these compounds may increase the risk of metal leaching from pipes into water when used without corrosion mitigation measures [20,22,23].

Research has shown that low-income, minority communities have a disparate risk of lead exposure through ingestion or inhalation of lead-based paint, dirt, or fumes compared to more affluent populations. However, few studies have explored the environmental justice inequities related to lead in drinking water, particularly at the community level. In this short communication, we assessed lead levels in drinking water within a vulnerable community in Harris County, Texas, identified community concerns over water quality, and provided additional evidence to further examine the potential relationship between the occurrences of lead contaminated drinking water among vulnerable populations.

## 2. Materials and Methods

### 2.1. Study Location and Population

**Figure 1:**
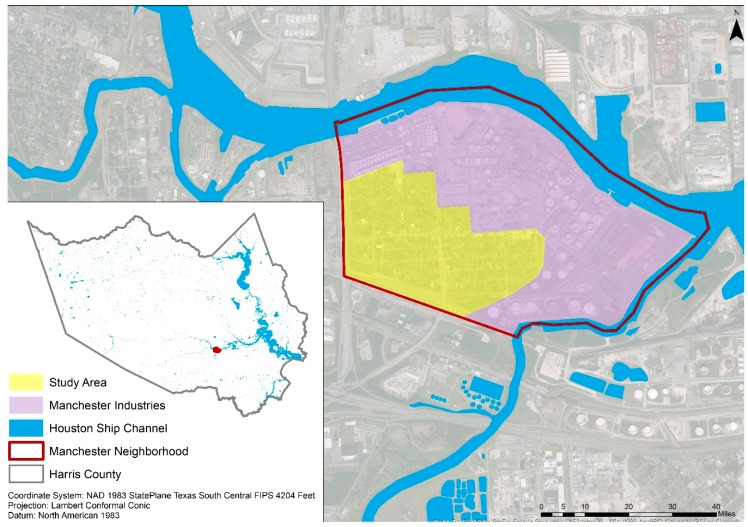
Location of the Manchester neighborhood in Houston, Texas.

Manchester is a small neighborhood located on the Houston Ship Channel in southeastern Houston, Texas (Figure 1), challenged by numerous issues related to flooding, air pollution, and health concerns [24,25,26]. The neighborhood lies in the close vicinity of 21 facilities that report to the EPA’s Toxic Release Inventory: Eleven of which are large quantity generators of hazardous waste, four treat, store, or dispose of hazardous wastes, nine major dischargers of air pollution, and eight major storm water discharging facilities [27]. Further, this neighborhood, along with other Houston Ship Channel communities, receives its drinking water from surface water sources and the City of Houston utilizes chloramines for water purification [28].

Houston is separated into 88 Super Neighborhoods, Manchester falls within super neighborhood 65, in which 98% of the population are minority. Manchester has a median income that is one-third less than the City of Houston overall and only six percent of residents have obtained a Bachelor’s degree [29]. Floodplains along the Sims Bayou have increased by 15 percent since 1980, due to increases in development and impervious cover like concrete and asphalt, while expected sea-level rise could expose another 35,000 residents in Ship Channel neighborhoods to flooding [30].

### 2.2. Household Survey

Three field teams consisting of at least one field-trained graduate student from the EpiAssist program at Texas A&M University [31] and at least one Spanish-speaker were deployed in February 2019 for data collection. Each of the three field teams was assigned 5 randomly-selected residences in the neighborhood of Manchester to attempt contact. Substitution with an adjacent residence was permitted if contact was made and no resident aged 18 years or above consented to participate or if the selected residence was inaccessible, had signage precluding entry, appeared to be abandoned, or was considered to be unsafe. A nine-question survey instrument was used to ascertain respondent demographics (gender, race, and age), water-use behavior, drinking water sources, and perceived quality and safety of household tap water. The survey instrument and accompanying informed consent materials were approved by the Texas A&M University Institutional Review Board (#0698D).

### 2.3. Household Tap Water

EPA Method 1694 was followed for water collection and transport [32]. Respondents to the household survey were asked to collect and provide tap water the following morning. Those who consented were provided a $10 Walmart gift card, equipped with sampling and packaging materials, and trained in the appropriate method for tap water sampling and storage. Specifically, participants were instructed to aseptically don nitrile gloves, collect 250 mL polypropylene laboratory containers at first draw from the kitchen faucet, then transfer the sealed container into the provided Styrofoam cooler (Polar Tech, Genoa, IL, USA). At 8:00 the following day, research teams returned to retrieve collected samples and sampling supplies. The collected samples were transported to a laboratory located in Houston, Texas, that is accredited by the National Environmental Laboratory Accreditation Program (NELAP) for analysis.

Tap water samples were assayed for lead concentrations via inductively coupled plasma-atomic emission spectrometry in accordance with EPA Method 200.7 [33]. Quality control was assured through the use of laboratory blanks, laboratory control samples, and sample duplicates (LCS/LCSD). In addition, matrix spike and spike duplicate (MS/MSD) were used for all samples. Tap water samples were also evaluated with the Langelier Saturation Index (LSI), which provides an indication of whether each water sample is likely to deposit minerals within the water system.

## 3. Results and Discussion

### 3.1. Household Survey

Of the 38 residences approached, contact was made at 18 households and 13 surveys were completed for a contact rate of 47.4% and a response rate of 72.2%. All surveys (N = 13) were completed by Hispanic or Latino individuals, 53.8% of whom were female (N = 7), 15.4% were male (N = 2), and 30.8% (N = 4) declined to report their gender (Table 1). Spanish was the preferred language for a majority of the respondents (76.9%; N = 10). Children (under 18 years of age) lived in 53.8% (N = 7) of the homes who participated.

The survey results allowed for the determination of whether or not residents held concerns over the quality of drinking water within Manchester (Table 2). Overall, while 30.8% (N = 4) of respondents indicated having concerns over the water’s quality, a majority of respondents (69.2%; N = 9) did not. A similar trend was observed when respondents were asked about specific issues. Only 7.7% (N = 1), 23.1% (N = 3), 23.1% (N = 3), and 15.4% (N = 2) of respondents reported concerns over the water being contaminated, having a bad smell, appearing cloudy or dirty, and being unhealthy, respectively. It was revealed that 75% of individuals who had concerns with the quality of their drinking water had the presence of lead in their water

### 3.2. Household Tap Water

Results of the water quality sampling indicated concentrations of lead in 30.8% (N = 4) of the homes sampled (Table 3). Further, of the homes with lead in the drinking water, 25% had children under the age of 18 living fulltime within the house. The levels of lead in the samples did not exceed the EPA’s action level of 15 parts per billion (µg/L) [34]. It is important to note that according to the CDC, no safe blood level has been identified for young children [35]. The EPA has stated that due to the potential of harmful effects even at low exposure levels, the contaminant level goal for lead in drinking water is zero [4]. Also of note, of the households who expressed concern that their water was contaminated with lead, 75% (N = 3) were correct.

### 3.3. Discussion

While much attention has been placed upon the failures of the city of Flint, Michigan [36,37], to properly treat its municipal system after a change in the source of water, research is now suggesting this is but one incident in a growing list of communities experiencing lead contaminated water. Our small pilot study was designed to evaluate perceptions of drinking water quality and safety among community members within the neighborhood of Manchester in Houston, Texas, and to assess the existence and extent of lead contamination in residential tap water. The findings from this research suggest that community members are concerned about potential hazardous exposures in their environment and points to a potential broader issue among all Houston Ship Channel residents.

There is a growing concern amongst our community partners, the Texas Environmental Justice Advocacy Services (t.e.j.a.s.), and the Green Ambassadors of Furr High School, as well as public health officials, that the poor environmental conditions documented within Manchester will be shown to exist across all Houston Ship Channel communities [38,39]. These concerns are informed by the reality that many of the households in these communities reside in older infrastructure, are composed of vulnerable population groups, and receive their water from the same sources and purification methods as Manchester. As the lead concentrations observed were below federal actionable levels, the decision to retrofit residential water infrastructure must be made and financed by community members. While this project benefitted from a strong response rate and utilization of known analytical methods, it was greatly limited from its small size. Another limitation of this project is a lack of data from other Ship Channel neighborhoods as well as not receiving multiple samples from Manchester or possible lead exposures from other sources.

## 4. Conclusions

Monitoring at the point of consumption needs to be expanded upon to ensure the health of residents across the nation. Additional research on the scope and breadth of lead contamination needs to be undertaken to establish the public health implications on individuals, especially vulnerable groups. Further, the confirmation of residents’ concerns of environmental quality may indicate that the experience of Manchester residents is not unique and is a common reality for those in other US communities characterized by environmental justice issues.

## Figures and Tables

**Table 1:** Sample characteristics.

Characteristics	N (%)
**Gender**	
Male	2 (15.4%)
Female	7 (53.8%)
Prefer not to answer	4 (30.8%)
**Race and Ethnicity**	
Non-Hispanic White	0 (0%)
Hispanic or Latino	13 (100%)
African American	0 (0%)
Other	0 (0%)
**Age (years)**	
Mean (SD)	53 (21.68)
**Age in Groups (years)**	
<35	2 (22.2%)
36–50	3 (33.3%)
51–69	2 (22.2%)
70+	2 (22.2%)
**Language Preference**	
Spanish	10 (76.9%)
English	3 (23.1%)

**Table 2:** Total number and percent of concerned respondents in the neighborhood of Manchester, Houston, Texas, by issue.

	N	%
**Tap Water Quality Concern**		
Yes, concerned	4	30.8
No, not concerned	9	69.2
**Perceived Tap Water Problems among Concerned**		
The water is contaminated	1	7.7
The water has a bad smell	3	23.1
The water looks cloudy/dirty	3	23.1
The water is unhealthy	2	15.4

**Table 3:** Lead concentrations (µg/L) in 13 tap water samples collected from residences located in the neighborhood of Manchester, Houston, Texas.

Sample No	1	2	3	4	5	6	7	8	9	10	11	12	13
**Lead (µg/L)**		1.2	2.4					1.0					0.6
